# *Mycoplasma genitalium* in Singapore is associated with *Chlamydia trachomatis* infection and displays high macrolide and Fluoroquinolone resistance rates

**DOI:** 10.1186/s12879-020-05019-1

**Published:** 2020-04-28

**Authors:** Tim Hart, Wen Ying Tang, Siti Aminah bte Mansoor, Martin T. W. Chio, Timothy Barkham

**Affiliations:** 1 Tan Tock Seng Hospital, 11 Jalan Tan Tock Seng, Singapore, 308433 Singapore; 2 Department of Sexually Transmitted Infections Control Clinic, 31 Kelantan Ln, Singapore, 200031 Singapore

**Keywords:** *Mycoplasma genitalium*, *Chlamydia trachomatis*, *Neisseria gonorrhoea*, Sexually transmitted diseases, Antibiotic resistance, Singapore

## Abstract

**Background:**

*Mycoplasma genitalium* is an emerging sexually transmitted infection, with increasing rates of resistance to fluroquinolones and macrolides, the recommended treatments. Despite this, *M. genitalium* is not part of routine screening for Sexually Transmitted Infections (STIs) in many countries and the prevalence of infection and patterns of disease remain to be determined in many populations. Such data is of particular importance in light of the reported rise in antibiotic resistance in *M. genitalium* isolates.

**Methods:**

Urine and urethral swab samples were collected from the primary public sexual health clinic in Singapore and tested for *C. trachomatis (CT)* or *N. gonorrhoeae* (NG) infection and for the presence of *M. genitalium*. Antibiotic resistance in *M. genitalium* strains detected was determined by screening for genomic mutations associated with macrolide and fluroquinolone resistance.

**Results:**

We report the results of a study into *M. genitalium* prevalence at the national sexual health clinic in Singapore. *M. genitalium* was heavily associated with CT infection (8.1% of cases), but present in only of 2.4% in CT negative cases and not independently linked to NG infection. Furthermore, we found high rates of resistance mutations to both macrolides (25%) and fluoroquinolones (37.5%) with a majority of resistant strains being dual-resistant. Resistance mutations were only found in strains from patients with CT co-infection.

**Conclusions:**

Our results support targeted screening of CT positive patients for *M. genitalium* as a cost-effective strategy to reduce the incidence of *M. genitalium* in the absence of comprehensive routine screening. The high rate of dual resistance also highlights the need to ensure the availability of alternative antibiotics for the treatment of multi-drug resistant *M. genitalium* isolates.

## Background

*Mycoplasma genitalium* is a sexually transmitted pathogen of rapidly increasing significance [1], which may cause cervicitis and urethritis in women and has been linked to pelvic inflammatory disease and infertility [2], as well as with non-gonococcal urethritis (NGU) in men [3]. Diagnosis of *M. genitalium* by bacterial culture is difficult due to requirements for specific media and prolonged incubation; in practice, diagnosis is constrained by a necessary reliance on molecular methods [4].

Treatment of *M. genitalium* has traditionally been with a single 1 g dose of azithromycin, using moxifloxacin as a second line agent. However, the recent increase in cases and the proportion of NGU caused by *M. genitalium* has been paralleled by a dramatic increase in resistance to both drug classes [5, 6], resulting in treatment failure [7]. *M. genitalium* is intrinsically resistant to beta-lactams and other antibiotics active at the cell wall, leaving realistic alternative treatment options limited. As quantitative antibiotic susceptibility tests are not widely performed due to the fastidious growth of the organism, monitoring of genetic markers of antibiotic resistance has become the mainstay of the clinical monitoring of *M. genitalium* susceptibility.

The significance of increased *M. genitalium* resistance is amplified in light of the commonality of co-infection with *Chlamydia trachomatis* (CT), a more established cause of urethritis. The standard first-line treatment of CT with single-dose oral azithromycin 1 g, has been shown to impact macrolide resistance in *M. genitalium* in co-infected patients [8]. With this in mind, we aimed to establish, for the first time, the prevalence of *M. genitalium* infection in Singapore, the degree of co-infection with CT, and levels of antibiotic resistance in these groups. Establishing these baselines is important to guide empirical therapy and will also allow us to gauge the rate of increase in, and significance of, resistance patterns in the future.

## Methods

All samples were taken from patients attending the DSC Clinic (Department of Sexually Transmitted Infections Control) in Singapore, with institutional ethical approval. The DSC clinic is the principle public specialist clinic for Sexually Transmitted Infections in Singapore. The protocol in DSC is to test for CT and NG together using either Urethral Swabs, or from urine samples. Left over samples were collected after routine clinically directed testing for CT and *Neisseria gonorrhoeae* (NG), with the Cobas 4800 CT/NG system (Roche), at the clinic during a one-month period between May and June 2016. PCR was used to test these samples for *M. genitalium*. Sequencing was used to detect mutations associated with resistance to macrolides and fluoroquinolones.

### M. Genitalium PCR

DNA was harvested from both positive and negative samples using automated extraction (Cobas 4800), followed by real-time PCR which simultaneously targeted both the *pdhD* and *mgpB* genes of *M. genitalium*. Primers and probes detecting the *pdhD* gene were adapted from those previously published by Müller [9]: MG-041 (5′-CGGATCAAGACCAAGATACTTAACTTT-3′), MG-042 (5′-AGCTTGGGTTGAGTCAATGATAAAC) and MG-048 (HEX-5′-CCAGGGTTTGAAAAAGCACAACAAGCTG-3′-BHQ1). Primers and probes detecting the mgpB gene were adapted from those used by Jensen [10]: MgPa-355F (GAGAAATACCTTGATGGTCAGCAA), MgPa-432R (GTTAATATCATATAAAGCTCTACCGTTGTTATC) and probe MgPa-380 (FAM-ACTTTGCAATCAGAAGGT-BHQ1). Both primer/probe sets were included in the same PCR reaction (12.5 μl SsoAdvanced™ Universal SYBR Green Supermix (Biorad), 5 μM each primer/probe set, 0.25 μl MG-048 probe (10 μM), 0.6 μl MgPa-380 probe (10μlM) and 5 μl DNA in a 25 μl reaction). PCR was carried out in a BioRad CFX96 machine with an initial denaturation of 95 °C for 3 min and followed by 45 cycles of 94 °C for 15 s and 60 °C for 30 s). Samples were considered positive for *M. genitalium* only when both PCR results were positive. Where only one target was positive, the PCR was repeated to ensure two positive results (this was necessary for 3 samples all of which had low Ct values for amplification and in all 3 a positive result was obtained on repeat PCR).

### Sequencing

23S rRNA gene sequencing of the 23S ribosomal RNA gene from 16 *M. genitalium* positive samples was performed as described previously [11]. The gene was amplified using F1-Mg (5′ GAAGGAGGTTAGCAATTTATTGC) and R1-Mg (5′ TTCTCTACATGGTGGTGTTTTG) and HotStar Taq (12.5 μl HotStar Taq Buffer (Qiagen), 1 μM each primer, 5 μl extracted DNA, in a 30 μl reaction). Sequencing was performed with PCR amplification primers and yielded around 150 nt sequence. Cycling conditions were 95 °C for 15 min and then 45 cycles of 94 °C for 15 s, 58 °C for 30 s, 72 °C for 30 s and a final elongation of 3 min at 72 °C. Sequences (GenBank accession MK411350-MK411365) were manually checked for the presence of alterations at nucleotide 2058 or 2058 (*E. coli* numbering).

*GyrA* or p*arC* sequencing was carried out as previously described [12] using either MG-GYRA-A (CGTCGTGTTCTTTATGGTGC) and MG-GYRA-B (ATAACGYYGTGCAGCAGGTC) primers or MG-PARC-A (TGGGCTTAAAACCCACCACT) and MG-PARC-B (CGGGTTTCTGTGTAACGCAT). HotStar Taq was again used, with 10 μM each primer and 5 μl extracted DNA (total 30 μl volume) and cycling was as for 23S rRNA sequencing. Comparison of the resulting 150 nucleotide sequences (GenBank accession MK411366- MK411397) with previously published mutations was through manual alignment.

## Results

Of 472 samples screened for CT and NG, 358 were urine samples and 114 urethral swabs. A total of 185 samples were found to be PCR positive for either CT, NG, or positive for both, with a total of 287 negative samples (Table 1). CT was found in 22.3% urine samples and 23.7% urethral swabs. NG was more frequently isolated from urethral swabs than urine samples, whether as the sole isolate (17.6% vs 11.7% respectively) or in combination with CT (6.1% vs 2.5%). However, overall there was no statistically significant difference between NG, CT or *M. genitalium* isolation from urethral swabs or urine.

**Table 1:** Detection of *M. genitalium,* and *C. trachomatis* or *N. gonorrhoeae* in Urine Samples (A) and Urethral Swabs (B) with respect to the presence (+ve) or absence (−ve) of *M. genitalium*. Percentages in parentheses refer to the proportion of total samples)

		*M. genitalium*		Total
		+ve	-ve	
**A**	**Urine**			
	CT	7 (2%)	73 (20.4%)	80 (22.3%)
	NG	0	42 (11.7%)	42 (11.7%)
	both	1 (0.3%)	8 (2.2%)	9 (2.5%)
	negative	5 (1.4%)	222 (62.0%)	227 (63.4%)
	total	13 (3.6)	345 (96.4)	358
**B**	**Swab**			
	CT	2 (1.8%)	25 (21.9%)	27 (23.7%)
	NG	0	20 (17.5%)	20 (17.5%)
	both	0	7 (6.1%)	7 (6.1%)
	negative	1 (0.9%)	59 (51.8%)	60 (52.6%)
	total	3 (2.6%)	111 (97.4%)	114

To estimate the relative rates of *M. genitalium* in CT and NG positive and negative samples, we randomly selected 184 samples from the 287 CT/NG negative samples and performed an *M. genitalium* PCR in comparison to the 185 CT/NG positive samples. Our results show an association of *M. genitalium* infection with CT co- infection (Table 2). The rate of *M. genitalium* infection in patients positive for CT was 8.1% (10/123, including a patient with both CT and NG) but *M. genitalium* was not detected in any of 62 patients with NG alone. The rate of *M. genitalium* in CT negative samples (including CT negative/NG positive samples) was 2.4% (6/246). This corresponds to an odds ratio of 3.54 for MG infection in patients with *C. trachomatis* infection as compared to *C. trachomatis* negative patients (*P* = 0.017, 95% Confidence Interval of 1.26 to 9.98).

**Table 2:** Prevalence of *M. genitalium* in *C. trachomatis* and *N. gonorrhoeae* positive and negative samples

*M. genitalium*	CT alone	NG alone	CT/NG Dual Infection^a^	CT/NG Negative	Total
positive	9 (7.3%)	0	1 (6.3%)	6 (3.3%)	16 (4.3%)
negative	98	62	15	178	353
Total	107	62	16	184	369

Percentage of positive samples are shown in parentheses

^a^Dual infection refers to samples in which both *C. trachomatis* and *N. gonorrhoeae* were detected

To determine macrolide and fluoroquinolone resistance rates in *M. genitalium*, we sequenced the genomic areas noted to be responsible for macrolide resistance (23S ribosomal RNA) [11, 13] and fluoroquinolone resistance (p*arC* or *gyrA* genes) [14]. 6 of our 16 *M. genitalium* isolates contained sequences previously associated with antibiotic resistance [14]. Interestingly, resistance mutations were exclusively found in those isolates which were also positive for *C. trachomatis* (Table 3A).

**Table 3:** Antibiotic resistance in *M. genitalium* exclusively found in *C. trachomatis* co-infected samples (A), with a high rate of co-resistance to macrolides (23S) and fluoroquinolones (parC) in the resistant isolates (B)

A					
		Wild-type	Resistance mutation		Total
	*C. trachomatis*	4	5		9
	*N. gonorrhoeae*	0	0		0
	CT/NG dual infection	0	1		1
	CT/NG negative	6	0		6
	Total	10	6		16
B					
		23S Mutation			
		wt	A2058G	A2059G	Total
parC mutation	wt	10			10
	Ser83 ➔Asn		1		1
	Asp87 ➔Tyr		1		1
	Ser84➔ Pro		1		1
	Ser83 ➔Ile	2		1	3
	Total	12	3	1	16

We found mutations in the 23S rRNA sequence (previously shown to confer macrolide resistance) in four of our *M. genitalium* positive samples (25%), and parC mutations (previously associated with fluoroquinolone resistance) in 6 samples (37.5%). The antibiotic resistance noted in our isolates is relatively diverse (Table 3B), demonstrating both of the mutations linked to macrolide resistance (nucleotides 2508 and 2509 of 23S rRNA [11]) and four different mutations in the *parC* gene associated with fluoroquinolone resistance [14] (Ser 83➔Asn, Ser84➔Pro, Asp87➔Tyr and Ser83➔Ile). Two thirds of the resistant isolates contained both fluoroquinolone and macrolide resistance mutations (Table 3B). No mutations were found in the *gyrA* gene and, other than a T➔A transition at position 1975 of the 23S gene of one isolate, there were no other mutations found.

## Discussion

Our study, the first of *M. genitalium* in Singapore, revealed strikingly different prevalences of *M. genitalium* depending on the presence of *Chlamydia trachomatis* co-infection: 2.4% *M. genitalium* positive samples in CT negative individuals and 8.1% in CT positive patients. The DSC clinic is both the major government sexual health clinic and the site of the mandatory screening of licenced commercial sex workers (CSWs) in Singapore. It is a relative weakness of this study that we do not have precise demographic details for our cohort, but they will have comprised both CSWs and non-CSW patients undergoing CT/NG testing for clinical urethritis. As such, our data would be expected to over-estimate the prevalence of *M. genitalium* in the general population in Singapore. Previously reported prevalences of *M. genitalium* have varied considerably depending on the population studied and our findings compare with rates as low 1.2% in the general UK population [15] and as high as 22.4% in adolescent women in the US [16]. Within studies based on CSWs, or in sexual health clinics, reported rates of *M. genitalium* prevalence tend to be higher than those found in our study, leading to a conclusion of a relatively low prevalence of *M. genitalium* in Singapore, even in those at risk of infection [17]. This fits a general pattern of lower rates of Sexually Transmitted Infections (STI) in Singapore than the global average, however, it should be noted that this is in the context of an overall increase of STIs in the country [18].

Our study was designed to determine the relative prevalence of *M. genitalium* in CT/NG infected and uninfected patients. Such a correlation has been noted in some studies [15, 16] but has been absent in others [19]. Our results show that, not only is there a heavy association of *M. genitalium* infection with CT infection in Singapore (odds ratio 3.5 as compared with CT PCR negative samples), but the odds ratio for this association (3.5) is much larger than previously published odds/hazard ratios of 1.9–2.5 [16, 20, 21]. While the association of CT and *M. genitalium* could result from shared risk factors for the transmission of these STIs, in our study, there was no association of *M. genitalium* with NG, which would also share similar risks for transmission, implying either a more a causal association between CT and *M. gentialium* infection, or at least a more nuanced epidemiological association.

The importance of considering CT co-infection while evaluating the burden of disease due to *M. genitalium* is highlighted by the concerning patterns of resistance in our samples. *M. genitalium* is very infrequently diagnosed in Singapore and thus very rarely specifically treated, whereas treatment of CT (or symptomatic urethritis) is common, with single-dose azithromycin. While single dose macrolide treatment was previously advocated for *M. genitalium,* evidence is growing to suggest that, in practise a five-day course of macrolides should be used to ensure *M. genitalium* eradication [7, 22]. Resistance mutations in our study were exclusively found in *M. genitalium* isolates taken from patients with CT co-infection. Although involving relatively small datasets due to the low prevalence of *M. genitalium* in Singapore, our data strongly supports a conclusion that a single dose macrolide is indeed non-curative with respect to *M. genitalium* and that the treatment of CT is, at least to some extent, driving *M. genitalium* resistance. In this respect, our study could be improved with clinical data on our patients’ past exposure to macrolides to definitively show that repeated treatment of serially acquired or recurrent CT infections, or use of macrolides or fluroquinolones to treat other infections, is associated with *M. genitalium* antibiotic resistance in Singapore. Our data is unusual in that resistance to macrolides is usually much higher than that of fluoroquinolones [19, 23–25], although predominance of fluoroquinolone resistance has previously also been noted [26, 27]. With respect to the latter issue, we noted that although fluoroquinolones are rarely used as first-line treatment of NGU in the DSC clinic in Singapore, we still note a high resistance to these infections in our sample. Such resistance may be due to a high frequency of antibiotic use for other conditions, such as respiratory or urinary tract infection. A high general antibiotic pressure from fluoroquinolones in Singapore is implied by recent findings of identical resistance mutations in the 23S rRNA of *M. pneumoniae* [28] in Singaporean patients.

The high rate of overall resistance (37.75% of our samples resistant to either fluoroquinolones or macrolides) and multi-drug resistance (62.25% of our resistant samples had resistance to both macrolides and fluoroquinolones) follows the global trend of increasing levels of macrolide and fluoroquinolone resistance in *M. genitalium* [6, 24, 26, 27]. The global situation of a slow, unnoticed accumulation of mutations to the only available therapies in patients with undiagnosed *M. genitalium* infection is concerning in light of the serious sequelae of untreated *M. genitalium.* The situation is compounded in Singapore, in which many of the newer antibiotics which have shown efficacy against *M. genitalium* are currently unavailable, in particular pristinamycin, a commonly recommended third line antibiotic [29]. Our results support the argument for close monitoring of antibiotic resistance in *M. genitalium* in Singapore, as in the rest of the world. Management of such resistance relies on timely diagnosis and appropriate treatment, ideally with subsequent confirmation of clearance of *M. genitalium* infections, but although diagnosis of *M. genitalium* has never been easier in light of novel and more convenient molecular tests [4], *M. genitalium* is often not part of routine screening or tested for in cases of NGU [8, 30]. Evidently the ideal approach would be to consider *M. genitalium* in all cases of NGU, routine screening and monitoring of populations at risk of high *M. genitalium* transmission, and susceptibility-guided therapy of treatment failures [31]. However, where this is not feasible, we believe that the close association of *M. genitalium* and CT in terms of infection and antibiotic resistance suggests that ensuring adequate diagnosis and treatment of *M. genitalium* in CT infected patients may constitute a more cost-effective approach in a situation of finite resources. Furthermore, in light of the high rates of (multi-drug) resistance in the *M. genitalium* noted in this study we agree with recently published guidelines [32] that such treatment should extend beyond a single dose. The high frequency of dual -resistant strains of *M. genitalium* in our study highlights the urgent need to make some of novel antibiotics, such as pristinamycin, which are considered third-line options in other countries, available in Singapore. Overall, our data, revealing a low but significant prevalence of *M. genitalium* in Singapore, associated with a high level of resistance to the available treatment choices, highlight the importance of developing a robust approach to diagnosis and treatment of *M. genitalium*, whether general or targeted, to avoid the emergence of untreatable *M. genitalium* in the region.

## Conclusions

This study has established some basic epidemiological benchmarks with respect to *M. genitalium* infection in Singapore. Our results point to a low prevalence in our study population, despite being based in a sexual health clinic, where prevalences are typically high; suggesting the potential to limit the emergence of *M. genitalium* in the country. Despite this, we found an alarmingly high level of resistance to the recommended first line antibiotics on a background of a low rate of screening for *M. genitalium* in the country. The strong association of *M. genitalium* with CT, for which there is more frequent testing, suggests that, at a minimum, patients with CT in Singapore should be screened for *M. genitalium.* Furthermore, treatment of CT (as well as other infections) should be undertaken with due consideration to the high resistance rates of *M. genitalium*. Our study highlights the need for a broader investigation of *M. genitalium* in Singapore and re-iterates the importance of ensuring that novel alternatives to macrolide and fluoroquinolone therapy are available.

## Data Availability

Sequences obtained in this study have been deposited in GenBank (Accession MK411350- MK411397). Any other data referenced in this report is available from the corresponding author on reasonable request.

## Abbreviations

CSW: Commercial Sex Worker
Ct: threshold cycle value
CT: *Chlamydia trachomatis*
DNA: deoxyribonucleic acid
DSC clinic: Department of Sexually Transmitted Infections Control clinic
NG: *Neisseria gonorrhoea*
NGU: non-gonorrhoeal urethritis
PCR: Polymerase Chain Reaction
rRNA: ribosomal Ribonucleic Acid
STI: Sexually Transmitted Infection
